# Open reduction and internal fixation for displaced Salter-Harris type II fractures of the distal tibia: a retrospective study of sixty-five cases in children

**DOI:** 10.1186/s13018-021-02359-9

**Published:** 2021-03-27

**Authors:** Quanwen Yuan, Yunfang Zhen, Zhixiong Guo, Fuyong Zhang, Jianfeng Fang, Zhenhua Zhu, Lunqing Zhu, Xiaofang Shen, Chunhua Yin, Yao Liu, Feng Yao, Lin Wu, Xiaodong Wang

**Affiliations:** Children’s Hospital of Soochow University, No.92 Zhongnan Street, Suzhou Industiral Park, Suzhou, Jiangsu province China

**Keywords:** Distal tibia physeal fracture, Salter-Harris type II fracture, Operative treatment, Premature physeal closure

## Abstract

**Background:**

The treatment for displaced Salter-Harris II (S-H II) distal tibia fractures remains controversial. The purpose of this study was to review S-H II distal tibia fractures and evaluate the rate of premature physeal closure (PPC) treated by open reduction and internal fixation (ORIF).

**Methods:**

We reviewed the charts and radiographs of S-H II fractures of the distal tibia with displacement > 3 mm between 2012 and 2019 treated by ORIF. Patients were followed up for a minimum of 6 months. CT scans of injured side or contralateral ankle radiograph were obtained if there was any evidence of PPC. Any angular deformity or shortening of the involved leg was documented. Multivariable logistic regression was performed to identify risk factors for the occurrence of PPC.

**Results:**

A total of 65 patients with a mean age of 11.8 years were included in this study. The mean initial displacement was 8.0 mm. All patients but one were treated within 7 days after injury and the mean interval was 3.7 days. Supination-external rotation injuries occurred in 50 patients, pronation-eversion external rotation in 13, and supination-plantar flexion in two. The residual gap was less than 1 mm in all patients following ORIF and all fractures healed within 4–6 weeks. Superficial skin infection developed in one patient. Ten patients complained of the cosmetic scar. The rate of PPC was 29.2% and two patients with PPC developed a varus deformity of the ankle. Patients with associated fibular fracture had 7 times greater odds of developing PPC. Age, gender, injured side, mechanism of injury, amount of initial displacement, interval from injury to surgery, or energy of injury did not significantly affect the rate of PPC.

**Conclusions:**

ORIF was an effective choice of treatment for S-H II distal tibia fractures with displacement > 3 mm to obtain a satisfactory reduction. PPC is a common complication following ORIF. The presence of concomitant fibula fracture was associated with PPC.

## Background

Salter-Harris type II (S-H II) distal tibia physeal fracture is the most frequent ankle injury in children, accounting for 32% to 60% [1–3]. Theoretically, S-H II distal tibia fracture carries a lower rate of growth disturbance, contributing to the fracture line being in the hypertrophic zone [4]. Previous literatures reported that the incidence of premature physeal closure (PPC) is 2% to 5% [5–7], based on the minimal displacement or relative shorter follow-up period. Recently, several articles have demonstrated a higher rate of PPC, ranged from 20 to 75%, regardless of the treatment options [1, 3, 8–12]. Moreover, PPC was far more common in S-H II fractures than in S-H III and IV fractures, representing up to 60% of all PPC [1, 2].

The optimal treatment for displaced S-H II distal tibia fractures is still controversial, even at the same institution [3, 8, 10]. Barmada et al. [3] reported that the incidence of PPC will increase by 3.5-fold in presence of residual gap, and they recommended open reduction and removal of the interposed periosteum to decrease the risk of PPC. However, Russo et al. [8] insisted that open reduction and internal fixation (ORIF) could not reduce the incidence of PPC. Spiegel et al. [13] categorized S-H II fractures of the distal tibia as unpredictable risk based upon the rate of complication. At our institution, S-H II fractures with displacement > 3 mm were reduced no more than once at the time of injury and ORIF was undergone to remove the interposed periosteum and to decrease the iatrogenic damage to the physis. The purpose of this study sought to review the S-H II distal tibia fractures with displacement > 3 mm and to evaluate the rate of PPC treated by ORIF.

## Methods

This retrospective study was approved by ethic committee board of our hospital. The medical records and radiographs of patients treated at our institution for S-H II fractures of the distal tibia with open physis were reviewed between 2012 and 2019. Patients with less than 1 year growth remaining (male > 15 years and female > 13 years), initial fracture displacement < 3 mm, pathological and open fractures, or < 6 months of follow-up were excluded. The parameters that were recorded included sex, age, side of injury, mechanism of injury, initial fracture displacement, the interval between the injury and surgery, and length of follow-up. The fracture displacement was measured in millimeters as the largest amount of displacement between the epiphysis and metaphysis on radiograph or computed tomography (CT). The mechanism of injury was classified based on Dias-Tachdjian system [14], including three fracture types: pronation-eversion external rotation (PER), supination-external rotation (SER), and supination-plantar flexion (SPF) injuries. We considered sports-related injuries and falls from < 1.0 m as low energy and motor vehicle accident, including e-bike accident, falls from ≥ 1.0 m as high energy.

Given severe displacement of the fracture, open reduction was performed under general anesthesia to obtain an anatomic reduction. At the time of surgery, the anterolateral approach approximately 3 to 5 cm was made and the periosteum and other soft tissue interposed in the physeal separation were removed prior to reduction, as described by Mosca [15]. The reduction was obtained via gentle manipulation reversing the direction of the original force that caused the injury. Following reduction, fixation was performed with either 2–3 1.6 mm smooth Kirschner wires (K-wire) that crossed the physis percutaneously or screws placed in the metaphysis 0.5–1.0 cm to the physis (parallel to the physis). The fibular fracture was fixed with plate and screws or K-wire only for overweight patients. A short leg cast was applied, which was retained for approximately 4 to 6 weeks. Hardware was removed routinely. The wires were removed 4–6 weeks and screws after approximately six months postoperatively. At each visit, anteroposterior (AP) and lateral radiographies were taken to recognize PPC. CT scans of injured side or contralateral ankle radiograph were obtained if there was any evidence of PPC. Any angular deformity or shortening of the involved leg was documented.

Descriptive statistics including means and frequencies were calculated for each of the examined variables. Chi-square analysis was used for categorical variables and Student’s *t* test was performed to compare continuous variables. Multivariable logistic regression was performed to identify the independent predictors of PPC. Data were analyzed using SPSS software, version 20.0 (SPSS Inc., Chicago, IL). A *P* value < 0.05 was considered statistically significant.

## Results

Sixty-five patients, 47 boys and 18 girls, were included for review. The average age was 11.8 years (range, 3.9 to 14 years). Thirty-eight fractures involved the right leg and 27 the left. The mean initial displacement was 8.0 mm (range, 3.2–25 mm). Forty-two patients had concomitant fibula fractures and 23 did not. SER injuries occurred in 50 patients, PER in 13, and SPF in two. There were 43 patients suffering from low-energy injury and 22 high-energy injuries. Four patients underwent surgery within one day after injury, 60 between 2 and 7 days, and one on the ninth. The mean interval was 3.7 days (range, 1–9 days). The mean follow-up period was 8.7 months (range, 6–25 months).

**Fig. 1:**
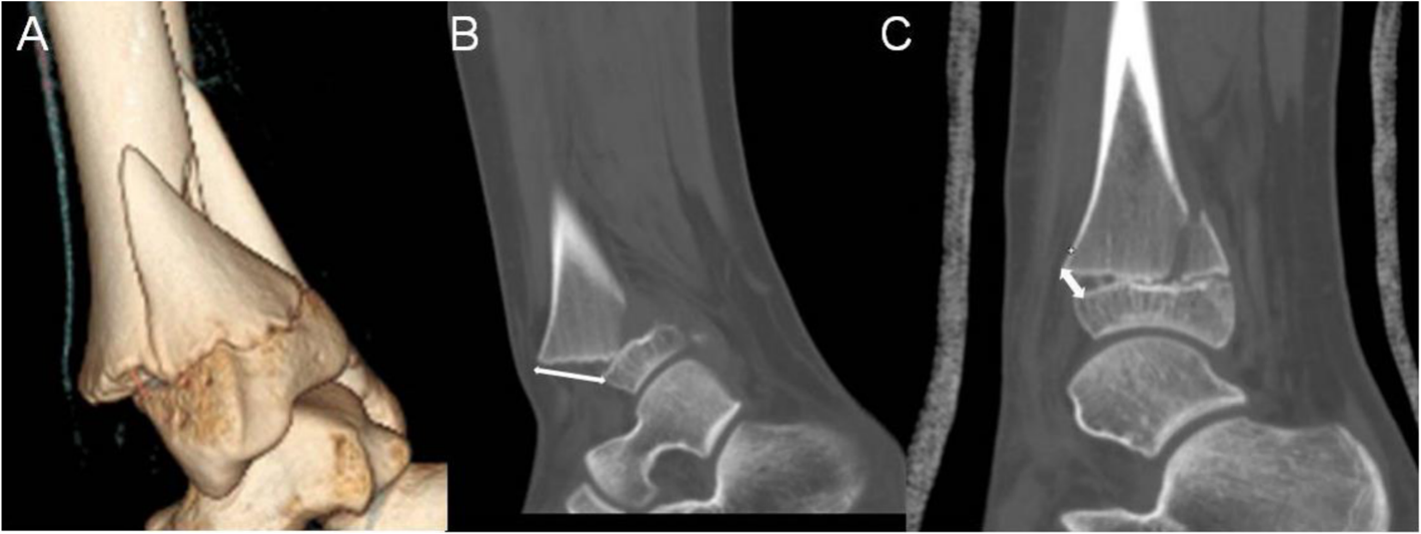
A 13-year-old boy fell downstairs and suffered a twist to his right ankle. **a** 3D CT view showed a displaced S-H II distal tibia fracture. **b** The maximal distance of displacement of the fracture was 18 mm (white double arrow) demonstrated in sagittal view. **c** The residual displacement was approximate 5 mm after closed reduction and casting

**Fig. 2:**
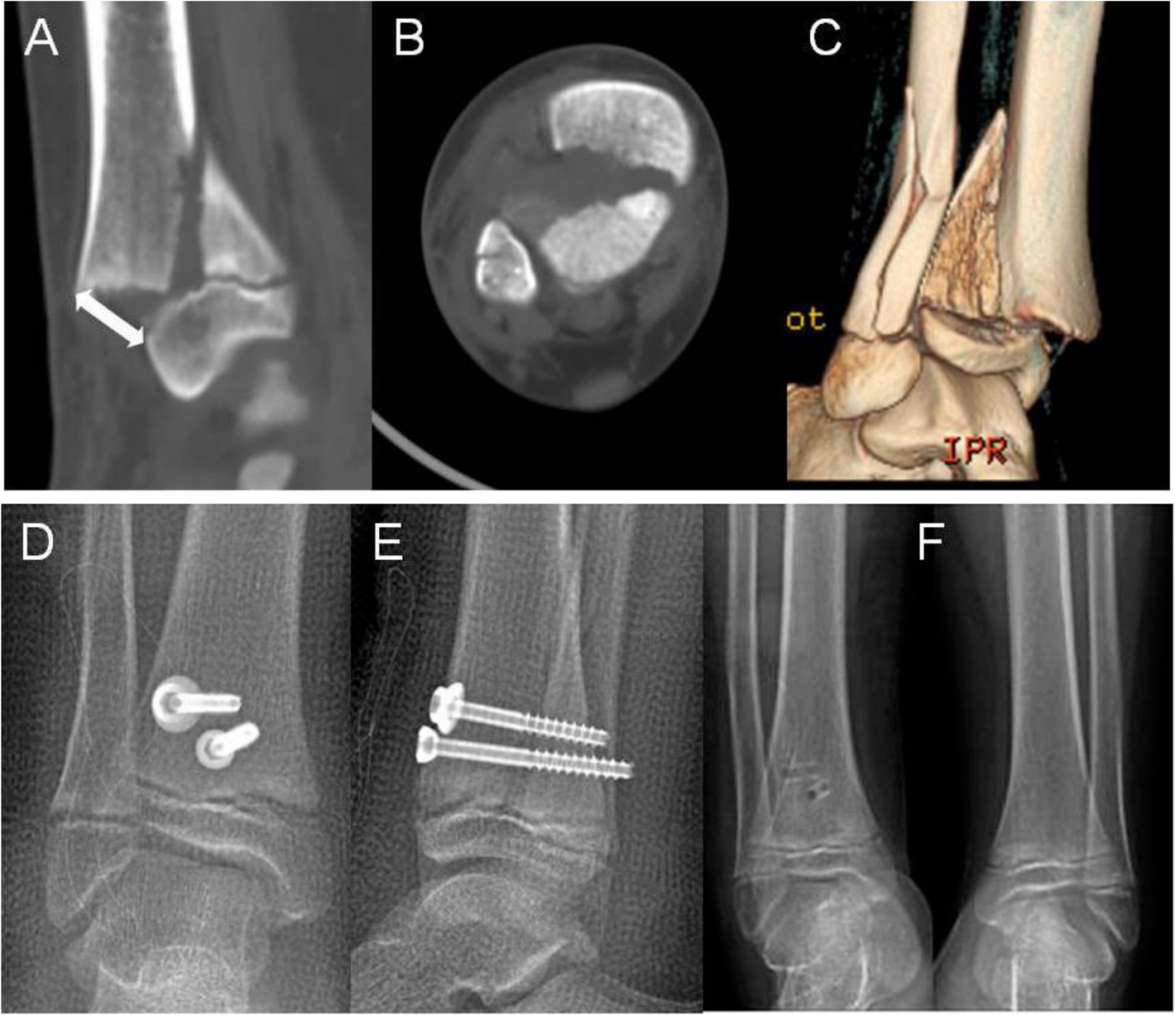
A 12-year-old boy. **a**–**c**, Sagittal, axial and 3D view demonstrated 15 mm displacement of the fracture (white double arrow ). **d**, **e** AP and lateral radiographs demonstrated the anatomical reduction immediately post operatively. **f** AP radiograph of bilateral ankle joints taken at 9-month follow-up showed symmetrically open physis as compared with the opposite side

**Fig. 3:**
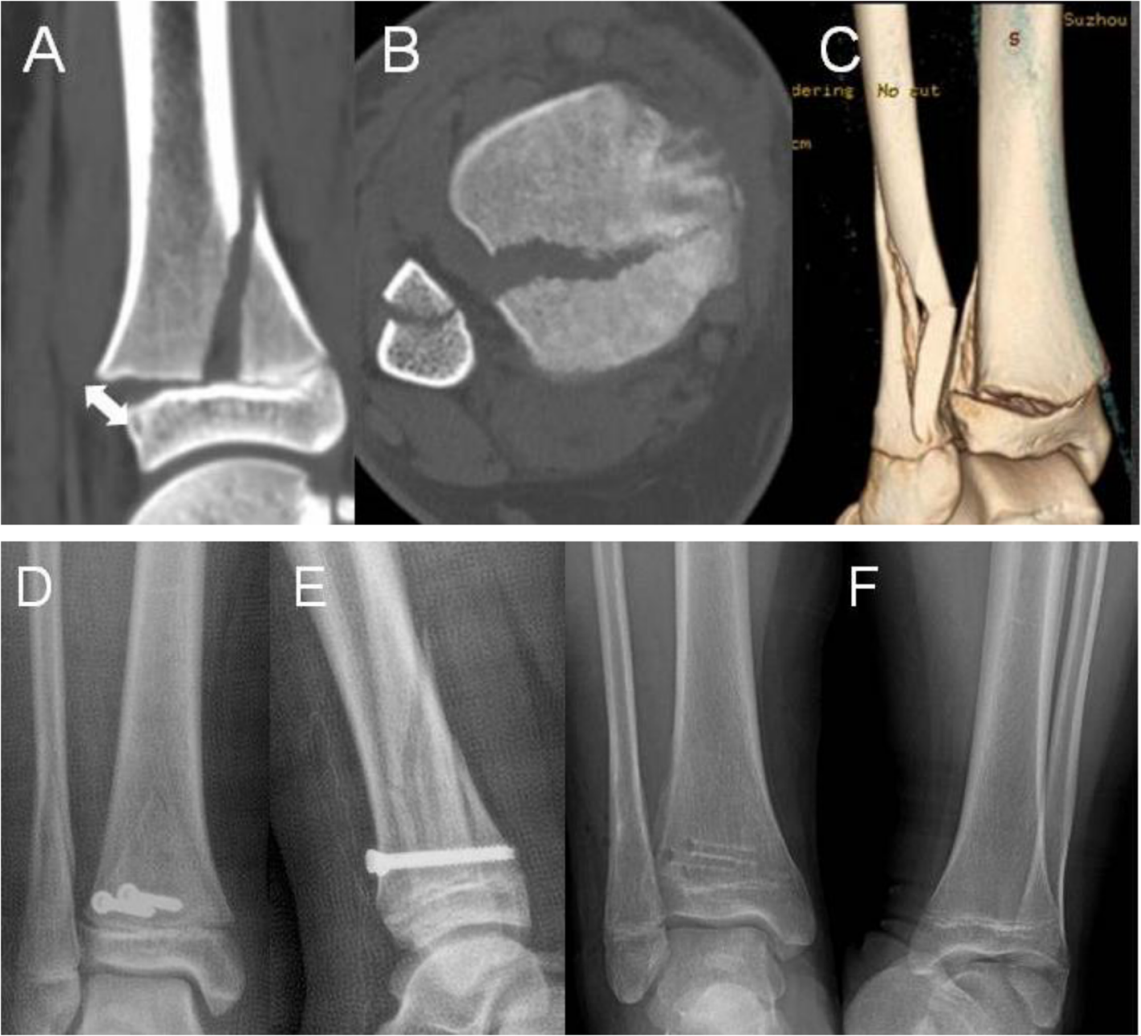
A 13-year-old boy. **a**–**c**, Sagittal, axial and 3D view demonstrated 8 mm displacement of the fracture (white double arrow). **d**, **e** AP and lateral radiographs demonstrated the anatomical reduction immediately post operatively. **f** AP radiograph of bilateral ankles obtained 7 months after ORIF showed PPC without any ankle deformity

All seventeen patients, in whom X-ray or CT scan was obtained immediately after closed reduction, had residual displacement of > 3 mm (Fig. 1). The residual gap was less than 1 mm in all of 65 patients following ORIF (Figs. 2 and 3). The presence of interposed periosteum was found in all patients but one. All fractures healed within 4–6 weeks. None of the patients suffered deep skin infection and one superficial skin infection treated with oral antibiotics. Ten patients complained of the cosmetic scar.

**Fig. 4:**
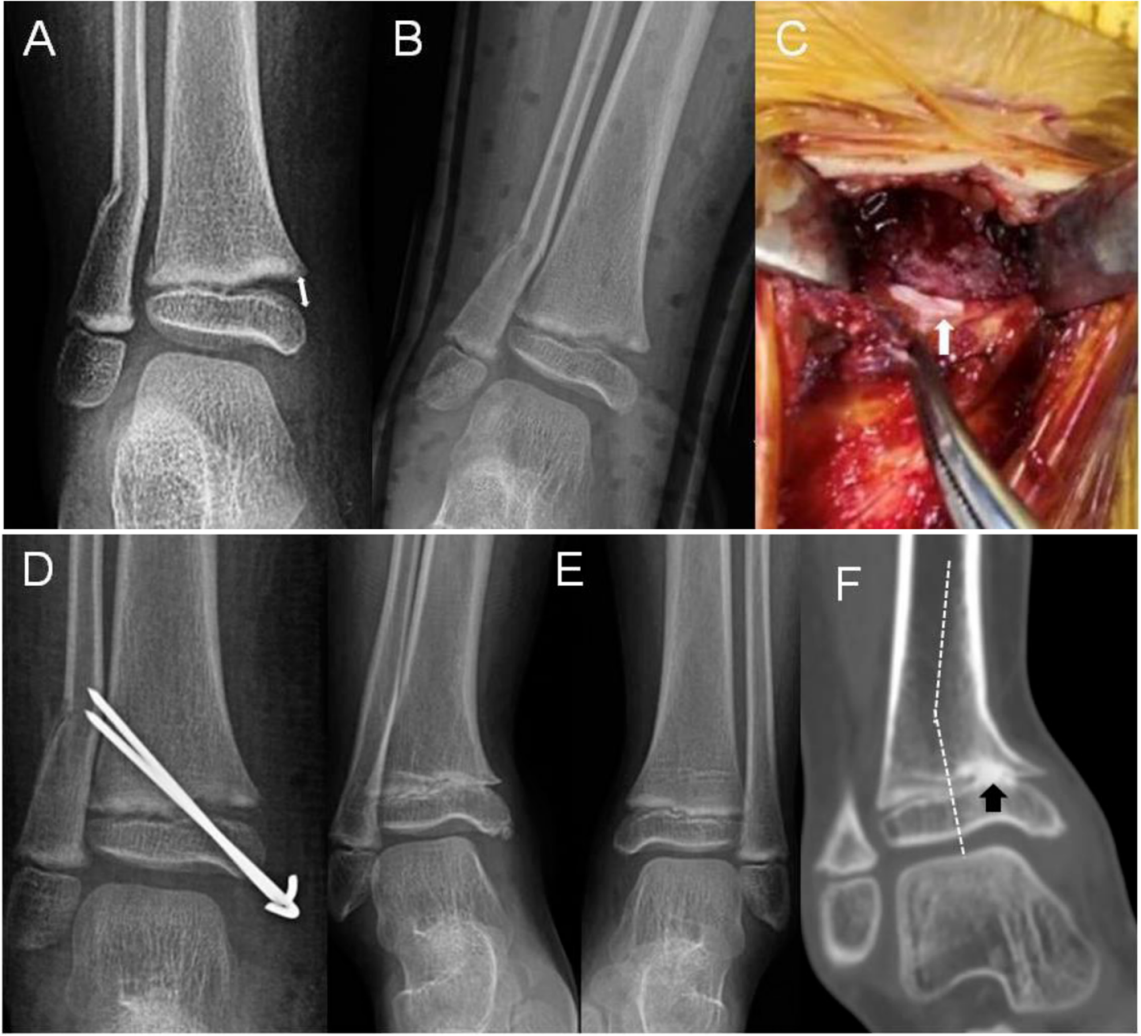
A 3.9-year-old girl. **a** AP radiograph showed 7 mm displacement of the fracture (white double arrow). **b** AP radiograph obtained 8 days after closed reduction showed the significant residual gap. **c** Interposed periosteum was shown after open reduction (white arrow). **d** AP radiograph demonstrated the reduction immediately post operatively. **e** AP radiograph of bilateral ankles obtained 14 months after treatment showed a partial premature arrest along the medial tibial physis. **f** Coronal view detected the bone bridge (black arrow) and a varus deformity of 15 degrees (white dashed line)

At finial visit, nineteen patients developed PPC and the rate of PPC was 29.2% (Fig. 2). Out of the 19 patients with PPC, two had a varus deformity of the ankle (10 and 15 degrees) (Fig. 4). In those patients who developed PPC, the average length between time of injury and time to diagnosis was 6.9 months (range, 2 to 12 months).

Variables associated with PPC were evaluated. When comparing patients with PPC and without PPC, the incidence of PPC was found to be significantly greater in patients with concomitant fibular fracture as compared with those with intact fibular (*P* = 0.001). However, there were no significant correlations between the PPC and patient age, gender, injured side, mechanism of injury (only SER vs PER), amount of initial displacement, interval from injury to surgery, and energy of injury (Table 1). The PPC rate by the interval was 50% within the first day, 18.3% between 2 and 7 days, 100% on the ninth, respectively. No statistical analysis was conducted due to the small sample. On multivariate logistic regression, concomitant fibular fracture was significantly associated with PPC. Patients with associated fibular fracture had 7 times greater odds of developing PPC (*P* = 0.01) (Table 2).

**Table 1:** Variables associated with PPC for the 65 patients with displaced S-H II distal tibia fractures

Variables	PPC (*n* = 19)	No PPC (*n* = 46)	*P*
Age (years)	11.5.2 ± 2.3	11.9 ± 2.0	0.52
Gender			
Female	5	13	0.87
Male	14	33	
Injured side			
Right	10	28	0.54
Left	9	18	
Mechanism of injury			
SER	14	36	0.89
ABD	4	9	
SPF	1	1	
Concomitant fibula fracture			
Yes	17	25	**0.001**
No	2	21	
Initial displacement (mm)	8.7 ± 3.2	7.7 ± 4.2	0.31
Interval (days)	3.6 ± 2.1	3.7 ± 1.6	0.93
Energy of injury			
High	6	16	0.81
Low	13	30	
Follow-up (m)	10 ± 3.1	8.0 ± 3.4	0.05

*SER* supination-external rotation, *PER* pronation-eversion external rotation, *SPF* supination-plantar flexion, *PPC* premature physeal closure

**Table 2:** Multivariable logistic regression analysis of risk factors with PPC in 65 patients with displaced S-H II distal tibia fractures

Variables	OR	95% CI	*P*
Gender			
Female	0.91	0.24–3.5	0.89
Male			
Injured side			
Left	1.61	0.48–5.42	0.44
Right			
Mechanism of injury			
SER	0.85	0.16–4.58	0.85
PER			
Concomitant fibula fracture			
Yes	7.82	1.56–39.03	**0.01**
No			
Energy of injury			
High	0.64	0.15–2.72	0.54
Low			

*SER* supination-external rotation, *PER* pronation-eversion external rotation, *CI* confidence interval, *OR* odds ratio

Table 3 showed that there was significant association between concomitant fibula fracture and the amount of initial fracture displacement (*P* = 0.002).

**Table 3:** The correlation between the concomitant fibula fracture and energy of injury, the amount of initial fracture displacement

Variables	Concomitant fibular fracture (*n* = 42)	Intact fibula (*n* = 23)	*P*
Energy of injury			
High	16	6	0.338
Low	26	17	
Initial displacement (mm)	9.1 ± 4.4	6.0 ± 1.9	**0.002**

## Discussion

This study shows the results of operative treatment of displaced S-H II fractures of the distal tibia. PPC was detected in 29% of our patients and was associated with the concomitant fibular fracture when the displacement was greater than 3 mm at time of injury.

The quality of the reduction was related to the severity of the initial displacement [16]. Margalit et al. reported that up to 60% had residual displacement > 2 mm after closed reduction [11]. In our series, we found that all of the 17 patients in whom radiographs were achieved immediately after closed reduction had residual displacement > 3 mm. And we also observed that the periosteum was interposed in the anterolateral corner of the physis in all patients but one, as already observed by a recent magnetic resonance imaging study [17].

There has been no consistent standard for acceptable reduction or no consistent strategy of treatment to dramatically decrease complications. The indication for ORIF is still under discussion. Generally, a threshold of greater than 3 mm of postreduction displacement indicated that an open reduction was required [3]. In addition, some authors stressed the importance of clinical judgment in management decisions [2, 5]. Each millimeter of initial displacement and residual gaps (> 3 mm) in the physis following closed reduction may increase the risk of PPC [1, 3]. In light of significant residual displacement after closed reduction and the entrapped periosteum, we performed ORIF for patients with > 3 mm of initial displacement. Although perfect reduction was obtained following ORIF, this may increase the treatment of ORIF in terms of the cutoff above-mentioned. Moreover, more attention about the cosmetic scar and the additional surgery to remove the hardware be paid. But our study did not elucidate whether ORIF was necessary if the displacement was < 3 mm after closed reduction. Some authors have demonstrated that nonoperative treatment can lead to good results among various postreduction displacement [8, 11, 18, 19].

Previous studies have shown that multiple factors were responsible for PPC [1, 6, 9–11, 13, 18]. However, we only found significant association between PPC and concomitant fibular fracture, which was in accordance to previous literatures [2, 5, 20]. Dias and Tachdjian [14] emphasized that the force which caused the ankle injuries would sum and such injuries always occurred in a sequence of grades. In our study, greater amount displacement in patients with fibular fracture likely reflected more severe shearing damage to the growth plate. Several studies have found an increased rate of PPC in high-energy injury [1, 11]. Leary et al. [1] demonstrated that patients with high-energy injury had an incidence of 86% PPC versus 14% for low-energy injury ( *P* < 0.001 ). In our study, we found no significant correlation between PPC and energy of injury. This may be explained that the force causing the fractures with severe displacement among subgroups by degree of injury was sufficient to result in crushing physeal damage. In addition, this seems to partly explain the higher rate of PPC than anticipated following ORIF. Aitken [21] concluded that treatment cannot resolve the growth disturbance resulting from the crushing force at the time of injury.

In our study, the operation was delayed to 3.7 days after injury due to significant swelling of the ankle. Two patients, who were treated on the sixth and ninth day after injury, developed varus deformity of the ankle. Given the rarity of the deformity, we cannot conclude whether this isolated case was due to the delayed reduction or not. But we think the earlier the reduction, the lower rate of PPC.

A limitation of our study was less length of follow-up. The average length from injury to diagnosis of PPC was 7 months, even 2 years after injury [5, 8]. Therefore, the patients with S-H II distal tibia fractures should be followed until skeletal maturity to identify the PPC.

In conclusion, displaced S-H II distal tibia physeal fractures have a high risk of periosteal entrapment. ORIF is an effective choice of treatment to obtain an anatomical reduction for patients with > 3 mm of initial displacement. However, PPC is a common complication following ORIF. The presence of associated fibula fracture plays an important role in the fracture outcome.

## Data Availability

All data generated or analyzed during this study are included in this manuscript.

## Abbreviations

S-H II: Salter-Harris type II
PPC: Premature physeal closure
SER: Supination external rotation
PER: Pronation-eversion external rotation
SPF: Supination-plantar flexion
K-wire: Kirschner wire
ORIF: Open reduction and internal fixation
AP: Anteroposterior radiograph
CT: Computed tomography
CI: Confidence interval
OR: Odds ratio
